# Specific impact of cardiovascular risk factors on coronary microcirculation in patients with subclinical hypothyroidism

**DOI:** 10.5937/jomb0-34545

**Published:** 2022-07-29

**Authors:** Mirjana Stojković, Biljana Nedeljković-Beleslin, Milorad Tešić, Zoran Bukumirić, Jasmina Ćirić, Miloš Stojanović, Marija Miletić, Ana Đorđević-Dikić, Vojislav Giga, Branko Beleslin, Miloš Žarković

**Affiliations:** University of Belgrade, Faculty of Medicine, Belgrade; University Clinical Centre of Serbia, Clinic of Endocrinology, Diabetes and Metabolic Disease, Belgrade; University Clinical Centre of Serbia, Clinic for Cardiology, Belgrade; University of Belgrade, Faculty of Medicine, Instuitute of Medical Statistics and Informatics, Belgrade

**Keywords:** cardiovascular risk factors, coronary flow reserve, subclinical hypothyroidism, thyroid, kardiovaskularni faktori rizika, koronarna rezerva protoka, subklinička hipotireoza, štitasta žlezda

## Abstract

**Background:**

Although thyroid hormones have significant effect on cardiovascular system, the impact of subtle thyroid dysfunction such as subclinical hypothyroidism (SCH) remains to be determined. We investigated coronary flow reserve (CFR) in patients with subclinical hypothyroidism.

**Methods:**

Thirty two subjects with SCH and eighteen control subjects with normal serum thyroid hormones and thyroid-stimulating hormone (TSH) levels were included in the study. TSH, free thyroxine, free triiodothyronine, glucose, insulin, HbA1c, cholesterol, triglyceride and plasma levels of C-reactive protein were measured. Coronary diastolic peak flow velocities in left anterior descending coronary artery were measured at baseline and after adenosine infusion. CFR was calculated as the ratio of hyperemic to baseline diastolic peak velocity.

**Results:**

CFR values were not significantly different between the two groups (SCH 2.76±0.35 vs controls 2.76±0.42). There was a significant correlation of CFR with waist to hip ratio, hypertension, smoking habits, markers of glucose status (glucose level, HbA1c, insulin level, HOMA IR), cholesterol, LDL-cholesterol and triglyceride levels in SCH group, whereas only cholesterol level showed significant correlation with CFR in controls. There was no correlation between CFR and thyroid hormones.

**Conclusions:**

We concluded that there is a different impact of cardiovascular risk factors on CFR in SCH patients compared to healthy control and that these two groups behave differently in the same circumstances under the same risk factors. The basis for this difference could be that the altered thyroid axis "set point" changes the sensitivity of the microvasculature in patients with SCH to known risk factors.

## Abstract (sr)

**Uvod:**

Poznato je da tiroidni hormoni imaju značajan efekat na kardiovaskularni sistem, ali i dalje ostaje da se utvrdi uticaj suptilnih promena na nivou tiroidne osovine kao što je subklinička hipotireoza (SHT). Ispitivali smo koronarnu rezervu protoka (KRP) kod pacijenata sa subkliničkim hipotireoidizmom.

**Metode:**

Trideset dva ispitanika sa subkliničkom hipotireozom i osamnaest kontrolnih ispitanika sa urednim tiroidnim hormonskim statusom su bili uključeni u studiju. Mereni su TSH, fT4, fT3, glukoza, insulin, HbA1c, holesterol, trigliceridi i CRP. Koronarne dijastolne brzine protoka u levoj prednjoj silaznoj koronarnoj arteriji merene su na početku i nakon infuzije adenozina. Koronarna rezerva protoka je izračunata kao odnos hiperemijske i osnovne dijastolne brzine protoka.

**Rezultati:**

Vrednosti koronarne rezerve protoka se nisu značajno razlikovale između dve grupe (SHT 2,76 ± 0,35 u odnosu na kontrole 2,76 ± 0,42). Postojala je značajna korelacija KRP sa odnosom struka i kukova, hipertenzijom, navikama pušenja, markerima glikoregulacije (nivo glukoze, HbA1c, nivo insulina, HOMA IR), holesterolom, LDL holesterolom i nivoom triglicerida u SHT grupi, dok je samo nivo holesterola pokazao značajnu korelaciju sa KRP u kontrolnoj grupi. Nije bilo korelacije između KRP i hormona štitaste žlezde.

**Zaključak:**

Zaključili smo da postoji drugačiji uticaj kardiovaskularnih faktora rizika na koronarnu rezervu protoka kod pacijenata sa subkliničkom hipotireozom u poređenju sa zdravom kontrolom i da se ove dve grupe ponašaju različito u istim okolnostima, pod istim faktorima rizika. Osnova za ovu razliku mogla bi biti da promenjen "set point" tiroidne osovine menja osetljivost mikrovaskulature kod pacijenata sa SHT na poznate faktore rizika.

## Introduction

Subclinical hypothyroidism (SCH) is defined as mild elevation of thyroid-stimulating hormone (TSH) in the presence of normal free thyroxine (fT4) and free triiodothyronine (fT3) levels [1]. It is well known that overt hypothyroidism has negative impact on cardiovascular function [2][3]. The clinical importance of subclinical hypothyroidism in cardiovascular disease and mortality is still controversial, because of the inconsistent results [4][5][6], on the impact of SCH on cardiovascular function. Several studies including meta-analyses have suggested that there is an association between SCH and cardiovascular diseases [2][3][7][8][9][10], and that SCH is an independent risk factor for atherosclerosis and myocardial infarction in elderly women [11]. Razvi et al. [12] in their meta-analysis postulated that SCH is associated with increased cardiovascular morbidity and mortality only in younger subjects. On the other hand, in the last few years there were several studies that did not show relation between SCH and cardiovascular disease or cardiovascular and all-cause mortality [5][13][14]. Likewise, treatment of subclinical hypothyroidism in older persons did not show any clinical benefit [15].

Coronary flow velocity reserve (CFR) is defined as the ratio of hyperemic coronary blood flow velocity to baseline and reflects functional integrity of coronary microcirculation. It has been shown that reduced CFR is an early manifestation of atherosclerosis and coronary artery disease [16]. CFR measured by transthoracic Doppler echocardiography (TTDE) has an excellent correlation with CFR measured by positron emission tomography, which has been validated as a gold standard for noninvasive CFR measurement [17].

The present study was designed to investigate the impact on persistent SCH on the value of CFR, as assessed by transthoracic Doppler echocardiography, and consequently microcirculatory function.

## Materials and methods

The study group consisted of 32 patients with newly diagnosed persistent SCH (31 female, one male; mean age 52.6±14.8 years), and 18 healthy controls (17 female, one male; mean age 50.1±15.4 years). SCH was diagnosed on the basis of persistent TSH increase with free thyroid hormones level within the referent range. Patients were included in the study only if they had stable SCH which was demonstrated by repeated thyroid hormone profile after minimum four weeks. The institutional ethics committee approved the study protocol, and all participants signed informed consent to the study.

The exclusion criteria for SCH group as well as for the control group were history of coronary artery disease, valvular or congenital heart disease, cardiac rhythm abnormalities, diabetes mellitus, systemic, hepatic or renal diseases. Controls had a normal thyroid hormonal status.

All blood samples were collected between 08.00 and 09.00 h in the morning after overnight fast. Serum lipid levels (total cholesterol, high-density lipoprotein cholesterol, triglyceride), HbA1c and fasting glucose levels were measured using spectrophotometry commercial kits on an automatic analyzer c501 (Roche Diagnostics, GmbH, Mannheim, Germany). C-reactive protein values were analyzed by Immunoturbidimetric assay for the in vitro quantitative determination on a Cobas c501 analyzer (Roche Diagnostics, GmbH, Mannheim, Germany), using the latex-enhanced immunoturbidimetric assay. Low density lipoprotein cholesterol was calculated by Friedewald's formula. The serum TSH, fT4, fT3, TPOAb and insulin levels were measured using a electrochemiluminescence immunoassay (ECLIA) on the Roche Cobas e601 automated analyzer (Roche Diagnostics, Mannheim, Germany) and using a chemiluminescent microparticle immunoassay (CMIA) on an Alinity instrument (Abbott Diagnostics, Wiesbaden, Germany). Normal range for TSH was 0.27–4.2 mIU/L, for fT3 was 3.1–6.8 pmol/L, for fT4 12–22 pmol/L and for TPOAb 0–34 IU/mL). Body mass index (BMI), waist-to-hip ratio (WHR) and HOMA IR were also calculated, using standard formulas. Systolic and diastolic blood pressures (BP) were measured on the right arm of subjects in an upright sitting position after at least 5 min of rest using a sphygmomanometer.

CFR was performed using the Acuson Sequoia C 256 (Siemens Medical Solutions, Mountain View, CA, USA) with 4-MHz transducer. With the patient positioned in the left lateral decubitus, coronary flow was searched for in the mid/distal portion of the left anterior descending (LAD) coronary artery with the transducer placed at the cardiac apex or one intercostal space higher in order to obtain modified, three-chamber view. Color Doppler imaging was performed by decreasing the Nyquist limit to 1624 cm/s. With a sample volume 3–5 mm wide and positioned on the LAD color flow signal in diastole, pulsed Doppler tracings of peak flow velocities were recorded. After acquiring Doppler tracings in baseline conditions, under continuous echocardiographic monitoring, adenosine 140 mg/kg/min was administrated over 2 min and peak diastolic coronary flow velocities were obtained during maximal hyperemia. Three optimal flow profiles at rest and during hyperemia were obtained and results were averaged. CFR was calculated as the ratio of hyperemic to baseline diastolic flow velocities. Preserved CFR was defined as 2.0. All patients abstained from caffeine-containing drinks for at least 12 hours before the tests.

### Statistical analysis

Results were presented as mean ± standard deviation, frequency (percent) and median (range) in case of not normal distribution of data. Chi-square test was used to test differences between nominal data (frequencies). For parametric data independent samples t-test was used to test differences between groups. For numeric data with non-normal distribution and ordinal data Mann-Whitney U test was used. Chi-square test or Fisher's exact test were used to test differences between nominal data (frequencies). Correlation between the CFR for LAD as dependent variable and potential predictors was analyzed by linear regression. All p-values less than 0.05 were considered significant.

## Results

Age, gender, BMI, glucose, insulin levels, HOMA IR, cholesterol, HDL, LDL, levels, systolic and diastolic blood pressure, smoking habits were similar in SCH group and in controls. Triglyceride levels were higher in SCH group, whereas CRP also showed borderline higher values in SCH group. The fT3/fT4 ratio was significantly higher in SCH group, as well as titer of thyroid peroxidase (TPOAb) autoantibodies (Table 1).

**Table 1:** Patients characteristic BMI – body mass index; WHR – waist to hip ratio

	SCH group<br> (n=32)	Control group(n=18)	P
Age	52.6±14.8	50.1±15,4	0.509
Male/female	1/31	1/17	0.595
BMI	26.6±5.1	24.2±3.0	0.069
WHR	0.84±0.07	0.82±0,06	0.620
Hypertension (%)	40.6%	38.9%	0.904
Systolic BP (mmHg)	120.3±11.6	119.4±10.1	0.792
Diastolic BP (mmHg)	75.8±7.8	73.3±7.3	0.283
Smokers (%)	21.9%	22.2%	0.923
Glycose (mmol/L)	5.5±0.7	5.4±0.6	0.708
Insulin (mIU/L)	8.5 (1.4–23.5)	6.8 (1.4 –16.1)	0.983
HbA1c (%)	5.7±0.4	5.5±0.3	0.212
HOMA IR	1.8 (0.3–6.7)	1.6 (0.3-3.6)	0.861
Cholesterol (mmol/L)	5.60±1.02	5.63±1.08	0.914
HDL (mmol/L)	1.48±0.32	1.67±0.53	0.113
LDL (mmol/L)	3.47±0.85	3.46±0.70	0.986
Triglyceride (mmol/L)	1.30 (0.48–3.66)	0.97 (0.52–2.19)	0.044
CRP (nmol/L)	1.3(0.3–9.6)	0.7(0.1–2.1)	0.051
fT4 (mgl/L)	11.8±1.6	12.6±1.7	0.082
fT3 (pmol/L)	4.03±0.42	4.01±0.42	0.881
fT3/fT4	0.35±0.05	0.31±0.04	0.015
TSH (mIU/L)	7.70 (4.60–15.35)	2.08 (0.51–4.14)	<0.001
TPOAb (IU/mL)	248.5 (4–7413.5)	14.4(0.3–793.3)	<0.001

Baseline diastolic peak flow velocity (DPFV) of LAD was similar between the groups, as well as hyperemic DPFV. Accordingly, there was no statistically significant difference in CFR for LAD between the SCH group and the control group, and all the values in both groups were above the preserved limit of CFR (2.0), but with a wide range of scatterplot data (Table 2).

**Table 2:** Coronary flow velocity values in SCH and controls DPVF – diastolic peak flow velocity; CFR – coronary flow reserve;<br>LAD – left arterior descending coronary artery

	SCH group<br> (n =32)	Control group<br> (n=18)	P
Baseline DPVF of LAD (cm/s)	0.27±0.04	0.26±0.06	0.402
Hyperemic DPVF of LAD (cm/s)	0.75±0.18	0.70±0.17	0.379
CFR	2.76±0.35	2.76±0.42	0.999

By univariate linear regression analysis with CFR for LAD as dependent variable, CFR was inversely associated with the age and total cholesterol values in controls, whereas in SCH, CFR was related to the age, hypertension, smoking, total and LDL cholesterol, triglycerides, and glucose metabolism deterioration, and waist-hip ratio, implicating specific contributory effect of cardiovascular risk factors on CFR in patients with SCH (Table 3). There was no association between TSH level and CFR nor between fT4 level and CFR in both control and SCH group.

**Table 3:** Univariate linear regression with CFR for LAD as dependent variable

Variable	SCH group		Control group	
	B	p	B	p
Age	-0.012	0.003	-0.019	0.002
BMI	-0.013	0.282	-0.018	0.610
WHR	-2.090	0.013	-1.897	0.307
Hypertension	0.249	0.045	0.362	0.075
Systolic tension	-0.010	0.066	-0.013	0.192
Dyastolic tension	-0.010	0.231	-0.013	0.362
Smokers	-0.256	0.037	0.033	0.877
Glucose	-0.249	0.006	-0.311	0.063
HbA1c	-0.357	0.021	-0.547	0.168
Insulin	-0.023	0.024	-0.013	0.629
HOMA. IR	-0.102	0.009	-0.116	0.326
Cholesterol	-0.165	0.005	-0.179	0.056
HDL	-0.077	0.701	-0.334	0.082
LDL	-0.160	0.028	-0.166	0.153
Triglyceride	-0.195	0.020	0.146	0.496
CRP	-0.008	0.798	-0.043	0.815
fT4	0.038	0.337	-0.016	0.810
fT3	0.057	0.693	0.340	0.216
fT3/fT4	-0.790	0.496	4.873	0.150
TSH	0.009	0.698	0.121	0.169
TPOAb	<0.001	0.802	0.001	0.124

## Discussion

We have shown that in patients with SCH, microcirculatory function as assessed by 2D Doppler echocardiography derived CRF is generally preserved with wide scatter of data and without significant differences to patients with normal thyroid function. However, it seems that in patients with SCH, in comparison to normal thyroid function, the value of CFR is more dependent on traditional cardiovascular risk factors including hypertension, smoking, high cholesterol, and glucose metabolism deterioration. CFR by Doppler echocardiography, over last 10 years has been shown to be highly reproducible, efficacious and feasible noninvasive to assess microcirculatory dysfunction in different clinical scenarios affecting coronary microcirculation [18][19].

Based on a large number of studies and meta analyses conducted in the last twenty years, it is clear that SCH leads to a somewhat increased risk for cardiovascular disease, cardiovascular mortality and overall mortality [2][3][6][9][11][12][20][21], but the pathophysiologic mechanisms involved in this phenomenon are still to be defined.

Since subclinical hypothyroidism is a laboratory finding, the diagnosis of SCH should be made with caution. Different physiological conditions as well as other diseases can change the pituitary-thyroid axis, i.e. lead to a transient increase in TSH. There is also an increase in TSH with age, and this increase does not lead to increased cardiovascular mortality CVD [22]. One way to overcome such doubts is to prove persistently elevated TSH over a period of time, and as Hashimoto's thyroiditis is the most common cause of both overt and subclinical hypothyroidism [1], finding of elevated TPOAb could reinforce the diagnosis of SCH [23]. In our study the SCH group showed a significantly higher titer of TPOAb compared to the control group, thus confirming the existence of an autoimmune process in the thyroid gland in SCH group. We also confirmed persistently higher TSH values which, after initial elevated values, were confirmed by TSH redetermination. There was a significant increase in the fT3/fT4 ratio in the SCH group in our study, which we know to represent the adaptive mechanism of the thyroid axis due to increased activity of deiodinase 2 (D2), which mediates T4 to T3 conversion, as well as due to higher TSH-induced increase of T3 synthesis and secretion from the thyroid gland [24].

Of all anthropometric and biochemical parameters, only C-reactive protein (CRP) and triglyceride level were significantly higher in the SCH group than in the controls. This agrees with studies that have shown similar results [25][26]. CRP is known to be a strong independent risk factor for cardiovascular events [27], not only among those with stable and unstable angina [28] but also among individuals with no current evidence of cardiovascular disease [29]. Triglyceride level was also higher in SCH group in two large observational studies [30][31], and it is well known that elevated plasma triglyceride level is an independent risk factor for cardiovascular disease [32]. The values of cholesterol, its fractions and glycose related parameters (basal glucose level, insulin, HOMA IR) did not differ significantly between groups, which was also shown in some of the published papers [30][33], but there are also papers that show a significant difference between groups in relation to these parameters [34][35].

Only four studies have previously evaluated CFR in middle-aged patients with SCH [36][37][38][39]. The two of them used dipyridamole [36] and adenosine [37] as a stressor, and both adenosine and dipyridamole induce a hyperaemic stimulus that relaxes vascular smooth muscle cells in mostly endothel-independent way. In the third study, conducted by the Oflaz et al. [38] CFR for LAD was evaluated before and after the introduction of levothyroxine replacement therapy. The fourth study evaluated endothelial-mediated CRF in SCH subjects using cold pressor test to induce endothelium-dependent vasodilation [39]. Importantly, in comparison to previous studies our study did not find significant deterioration of CFR in SCH patients [36][37][39].

In particular, Baycan et al. [36] in 50 SCH patients and 30 controls (hyperaemia was induced by dipyridamole), showed no significant difference in anthropometric and biochemical parameters between the groups (BMI, lipids, CRP), but a significant deterioration of CFR due to blunted hyperaemic response in SCH group (2.38 ± 0.44 vs. 2.98 ± 0.47, p<0.0001) [36].

Oflaz et al. [37] with a smaller group of subjects (18 SCH, 24 controls) and adenosine as a stimulus of hyperaemia-endothelium independent vasodilation obtained similar results for CFR (SCH 1.97 ± 0.09 vs. controls 2.58 ± 0.08. The same authors evaluated CFR for LAD before and after the introduction of levothyroxine replacement therapy and showed that there was a significant increase in CFR for LAD in SCH group after six month levothyroxine substitution (2.03 ± 0.13 vs 2.54 ± 0.18) but the study was conducted on only ten patients with SCH [38].

Biondi et al. [39] also showed a significant difference in CFR between the SCH and control group (SCH 20, control 15), but they induced endothelium-dependent vasodilation and hyperaemia using a cold pressor test as an inducer (SCH 1.4 ± 0.2 vs. controls 1.9 ± 0.3 p< 0.0001) [39].

It is challenging to explain about the significant differences between our and previous results in CFR values, but few points should be emphasized regarding our study population, methodology and results. Our study population was older (SCH 52.6 ± 14, 8; controls 50.1 ± 15, 4) than study populations in Oflaz et al. [37] (SCH 45 ± 2; controls 48 ± 2 years), Baycan et al. [36] (41.4 ± 9.5; controls 41.3 ± 9.4 years) and Biondi et al. [39] (SCH38.4 ± 12.1; controls 41.4 ± 14.5 years), and since CRF is significantly negatively correlated with age, it is possible that the subtle vascular changes that might be detected in SCH are outweighed by changes due to aging. Further, in the study by Biondi et al. [39] CFR was measured after induction of endothelium-dependent vasodilatation, while in the remaining tree studies, including our study, endothelium-independent vaso-dilatation was induced. And third, all of these studies were performed on a relatively small number of subjects.

If we look at the dependence of CFR for LAD in our study, it is expected that in both groups there is a significant dependence of CFR on the age of the subjects. However, apart from age, there is only a significant dependence of CFR for LAD on total cholesterol in the control group. It is interesting, however, that in the SCH group, the dependence of CFR for LAD on several anthropometric and metabolic parameters (WHR, HTA, smoking, glycemia, HbA1c, basal insulin, HOMA IR, cholesterol, LDL, triglycerides) was obtained. These results suggest that individuals with subclinical hypothyroidism are more sensitive to certain metabolic, proatherogenic parameters, and this finding could be one of the explanations for the increased morbidity and mortality from cardiovascular disease in patients with SCH, which has been shown in several studies and meta-analyzes [2][3][9][10]. Since CRP is shown to be higher in patients with SCH compared to healthy controls, low but prolonged chronic inflammation could be the basis for greater sensitivity of the microvasculature in patients with SCH to other known cardiovascular risk factors and mechanism linking SCH and CVD, i.e. that SCH facilitate the effect of traditional risk factors on microvascular function.

### Study limitations

Our study reflects a single-center experience with a relatively small number of participants. Second, the cross-sectional design of our study limits its ability to establish causality between SCH and CFR, and long-term effects of SCH on microcirculatory and cardiovascular function.

## Conclusion

Our study has shown that people with subclinical hypothyroidism have a higher risk of chronic inflammation, which plays an important role in the development of atherogenesis and thus an increased risk of developing CHD. We also showed that in patients with SCH several known risk factors for atherogenesis have a significant impact on CFR for LAD which is not the case in the control group. Although we did not find a significant difference between groups in relation to CFR for LAD, the different impact of cardiovascular risk factors on CFR for LAD suggests that these two groups behave differently in the same circumstances under the same risk factors. The basis for this difference could be that the altered »set point« of the thyroid axis changes the sensitivity of the microvasculature in patients with SCH to known risk factors, making them more susceptible for low prolonged chronic inflammation. Further investigations on a larger number of participants are needed to address in depth the relation between SCH, CFR, chronic inflammation and cardiovascular risk factors.

## Dodatak

### Conflict of interest statement

All the authors declare that they have no conflict of interest in this work.

### List of abbreviations 

SCH, subclinical hypothyroidism;<br>CFR,coronary flow reserve;<br>LAD, left anterior descending coronary artery;<br>DPFV, diastolic peak flow velocity
